# Development and Performance Evaluation of a New Conformance Control Agent Gel

**DOI:** 10.3390/gels10100618

**Published:** 2024-09-26

**Authors:** Bin Ma, He Wang, Shu Jiang, Mengyu Chen, Lei Zhang

**Affiliations:** China University of Geosciences (Wuhan), Wuhan 430074, China; mbycsy@yeah.net (B.M.); jiangsu@cug.edu.cn (S.J.); mengyuchen1022@163.com (M.C.)

**Keywords:** supramolecular–polymer composite gel, double network, multi-scale fractures, injection performance, plugging strength

## Abstract

How to effectively plug the multi-scale fractured water channeling has always been the key to achieving efficient water flooding of fractured low-permeability oil reservoirs. In this paper, a new type of supramolecular–polymer composite gel is developed, which is suitable for plugging multi-scale fractured water channeling. The supramolecular–polymer composite gel is composed of a polymer (such as polyacrylamide), cross-linking agent (such as polyethyleneimine), supramolecular gel factor (such as cyclodextrin) and polarity regulator (such as ethyl alcohol). The mass fraction of polyacrylamide, polyethyleneimine, cyclodextrin and ethyl alcohol are 0.15%, 0.2%, 1% and 0.2%, respectively. At the initial state, the viscosity of the composite gelant system is less than 20 mPa·s. It has good injection performance in micro-scale fractures and can enter the deep part of a fractured reservoir. At 40 °C, the composite gelant system can form a gel with a double network structure after gelation. One of the networks is formed by the covalent interaction between polyacrylamide and polyethyleneimine, the other network is formed by the self-assembly of cyclodextrins under the action of the ethyl alcohol. The comprehensive performance of the composite gel is greatly improved. The strength of the composite gel is >5 × 10^4^ mPa·s, and it has good plugging strength in large-scale fractures. The composite gel can be used as a conformance control agent for fractured low-permeability oilfields.

## 1. Introduction

Fractured low-permeability oil reservoirs account for a significant proportion of oilfields in China, such as the Changqing oilfield and Yanchang oilfield with a capacity of tens of millions of tons [1,2,3,4]. In this type of oil reservoir, the micro-scale fractures are commonly developed. In addition, large-scale fractures are formed during the process of artificial fracturing to increase the production of the oil well. The presence of multi-scale fractures can significantly increase the initial oil production and quickly realize industrial exploitation [5,6,7,8]. However, after transferring to the development of water flooding, there is a high risk of fractured water channeling, causing the reservoir to transition from a low water cut stage to a high water cut stage in a short period of time, making it difficult to effectively exploit the large amount of remaining oil [9,10,11,12].

The conformance control technology to plug the fractured water channeling zone in deep oil reservoirs is the most reliable measurement for increasing oil production. To plug multi-scale fractured water channeling, the conformance control agents must enter deep reservoirs with good injection capability at the initial state. After gelation in the reservoirs, it must have high plugging strength to achieve deep fluid flow diversion [13,14,15,16,17]. However, it is difficult for the existing conformance control agents to simultaneously achieve the dual goals of injection capability in the micro-scale fractures and plugging capability in the large-scale fractures. The most widely used polymer gel system can be taken as an example. It is composed of polymer and crosslinking agent. At low concentrations, the gelant system has good injection capability when the polymer concentration is in the range of 1000–2000 mg/L, but the gelation strength is not enough. It is easy to break and it cannot plug the fractured water channeling [18,19,20,21,22]. Although increasing the concentration of the polymer can improve the gelation strength, it leads to an increase in the initial viscosity of the gelant system, which can easily result in the phenomenon of being unable to inject the fracture. Through the statistical analysis of the conformance control operation in some regions of the Changqing oilfield and Yanchang oilfield, it was found that 70% of operation failures were caused by this phenomenon. The imbalance between the injection capability and plugging strength of the conformance control agents severely restricts the effectiveness of conformance control [23,24,25,26]. To overcome this constraint, it is necessary to develop a new type of gel of conformance control agent with good injection performance and high plugging strength.

In recent years, supramolecular gel, as a new type of functional material, has received extensive attention. Supramolecular gel is formed by organic small molecules with specific functions, such as amino acids, cyclodextrins, etc., which can form a three-dimensional network structure through non-covalent bonding and self-assembly [27,28,29,30]. Generally, the molecular weight of the organic small molecules is less than 2000 Dalton. Because the composition unit is a small molecule, its aqueous solution has extremely low viscosity, and so it has unique advantages in injection capability. However, the high strength supramolecular gel requires a high concentration (>8 × 10^4^ mg/L), which makes its application cost unable to meet the requirements of oilfield production [31,32,33].

Based on the advantages and disadvantages of supramolecular gel and polymer gel, they are combined to achieve complementary advantages and synergy [34,35,36]. In this regard, research reports in fields such as biomedicine can provide some corresponding insights. For example, Wei et al. [37] reported a double network structure gel with excellent performance by compounding small molecule gel and polymer gel. The formed composite gel is of great significance for the controlled release of drugs. Zheng et al. [38] reported a smart polymer gel based on the action of small molecules. The formed gel not only has good mechanical properties, but also has excellent self-healing performance. When the gel is cut into two sections, it can achieve nearly 100% repair after 12 h. After the supramolecular gel and polymer gel are compounded, a double network structure gel with good shearing repair ability and high strength can be formed under their respective low concentration conditions. For the composite gel system, it not only retains good injection capability, but also significantly improves the overall performance. This shows that the supramolecular–polymer composite gel system has good injection capability, high gel forming strength and good shearing repair ability. However, this new type of composite gel system has only been studied and reported in recent years, and there is a lack of research reported on this type of composite gel as the conformance control and water shutoff agent. Therefore, the supramolecular–polymer composite gel system can be constructed as a potential conformance control agent for fractured low-permeability oil reservoirs.

## 2. Results and Discussion

### 2.1. Properties of Gels

**Figure 1:**
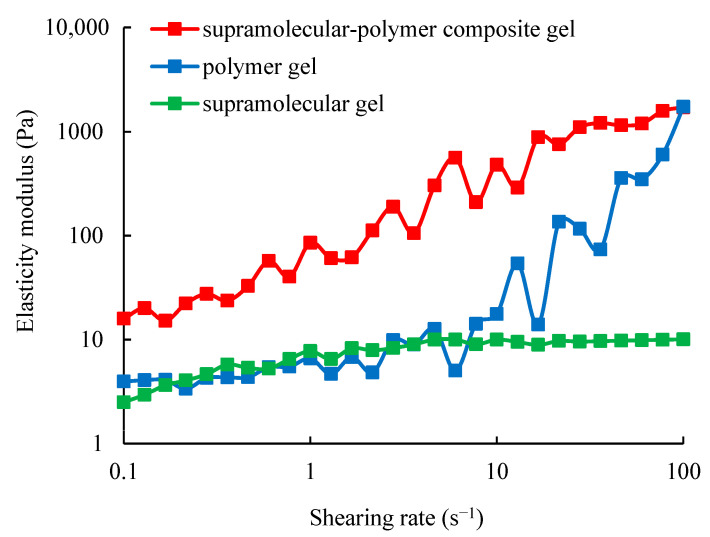
Variation curve of the elastic modulus of three gels with the shearing rate.

**Figure 2:**
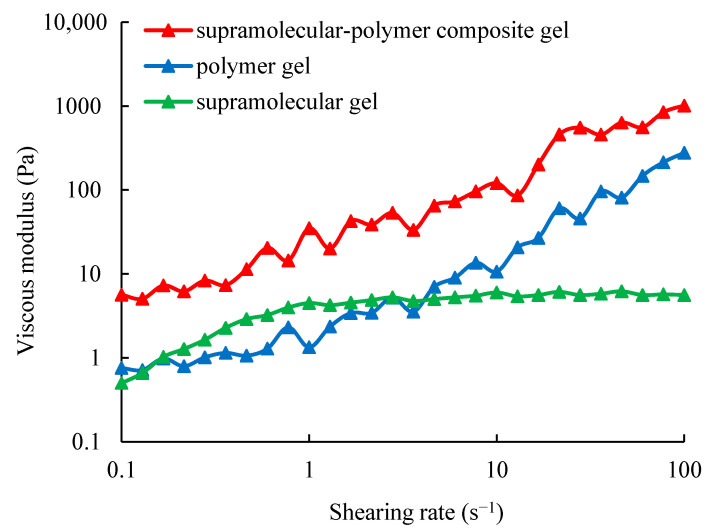
Variation curve of the viscosity modulus of three gels with the shearing rate.

For the supramolecular gel system at the initial state, its viscosity is 4.5 mPa·s, which is the same order of magnitude as water. After gelation, the viscosity is increased to 6000 mPa·s. The results of the viscoelasticity measurement are shown in Figure 1 and Figure 2. From Figure 1 and Figure 2, it can be observed that when the shearing rate is higher than 1 s^−1^, the elastic modulus and the viscous modulus of the formed gel no longer change with the shearing rate, and the elastic modulus is stable at about 9.8 Pa, while the viscous modulus is stable at about 5.6 Pa. The reason is that the formed supramolecular gel is a molecular aggregate by the action of the non-covalent bond, and this reversible non-covalent bond can achieve dynamic equilibrium under shearing [39,40]. Under shearing, non-covalent bonds can be instantly broken and then rapidly bonded to achieve dynamic equilibrium. Therefore, the elastic modulus and the viscous modulus of the supramolecular gel no longer change with the shearing rate.

For the polymer gel system at the initial state, the properties are basically the same as that of an aqueous polymer solution. Its viscosity is 17.5 mPa·s, which is one order of magnitude higher than that of water. After gelation, the viscosity is increased to 5800 mPa·s. The results of its viscoelasticity measurement are also shown in Figure 1 and Figure 2. From Figure 1 and Figure 2, it can be observed that the elastic modulus and viscous modulus of the polymer gel are both increased with the increase in the shearing rate, showing the typical viscoelastic material characteristics. This is because the polymer gel is formed by chemical bond cross-linking, and it is irreversible. As the shearing rate is increased, the polymer network needs to do more work to maintain its original form, resulting in an increase in its elastic modulus and viscosity model. The comparison between the polymer gel and the supramolecular gel shows that the viscoelasticity of the supramolecular gel is significantly different from that of the polymer gel.

For the supramolecular–polymer composite gel system at the initial state, the viscosity is 18.8 mPa·s, which is basically consistent with the initial viscosity of the polymer gel solution. This shows that the viscosity of the supramolecular–polymer composite gel is mainly controlled by the polymer. After gelation, the viscosity is increased to 54,000 mPa·s. It is much higher than that of the single polymer gel or the single supramolecular gel. This indicates that the combination of supramolecular and polymer materials has achieved even better results. The results of the viscoelasticity measurement are also shown in Figure 1 and Figure 2. From Figure 1 and Figure 2, it can be observed that the elastic modulus and viscous modulus of the formed composite gel are significantly higher than those of the single polymer gel or the single supramolecular gel, which indicates that the strength of the supramolecular–polymer composite gel is significantly increased. In addition, the viscoelastic characteristics of the three are different, and the root cause is the different network structures of the three.

### 2.2. Microstructure and Cross-Linking Reaction Mechanism of the Gels

**Figure 3:**
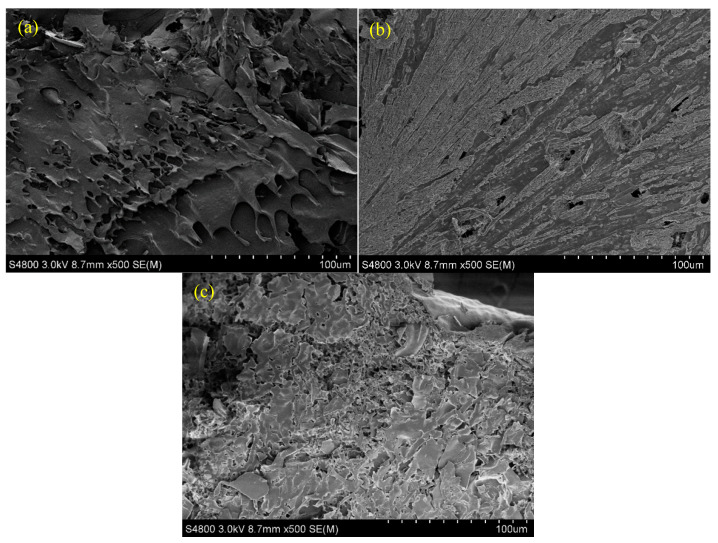
Microstructure of gels ((**a**): supramolecular gel; (**b**): polymer gel; and (**c**): supramolecular–polymer composite gel).

The results of the scanning electron microscope (SEM) of the microstructure of the gels are shown in Figure 3. Figure 3a shows the microstructure of the supramolecular gel. This appears to be a relatively fluffy and layered microstructure. Figure 3b shows the microstructure of the polymer gel. It presents the microstructure of a conventional linear polymer gel. Figure 3c shows the microstructure of the supramolecular–polymer double network gel. From Figure 3c, it can be observed that the gel has both a lamellar network structure and a wired network structure. For the supramolecular–polymer composite gel, the formed microstructure is denser and more compact, and the levels of microstructure are more distinct, which are significantly different from those of the polymer gel and the supramolecular gel.

**Figure 4:**
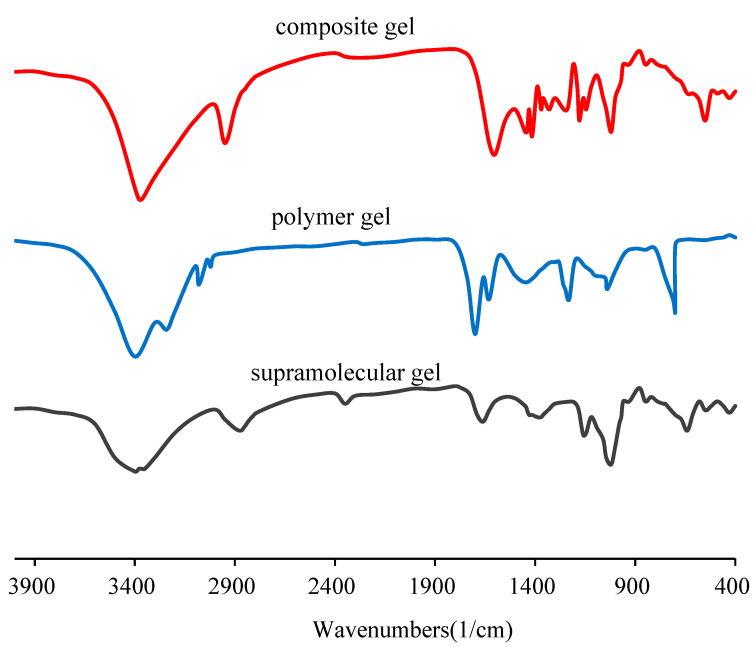
Infrared spectra of supramolecular gel, polymer gel and the composite gel.

The results of the infrared spectrum of the gels are shown in Figure 4. For the supramolecular gel, due to the ring vibration of cyclodextrin and the skeleton vibration of the α-1,4-glycosidic bonds of cyclodextrin, the two characteristic peaks appear at 640 cm^−1^ and 1045 cm^−1^, respectively. For the polymer gel comprising of polyacrylamide and polyethyleneimine, the stretching vibration peaks of carbonyl (C=O) groups and the bending vibration peak of N–H are observed at 1700 cm^−1^ and 1630 cm^−1^, respectively. The vibration peak at 1450 cm^−1^ corresponds to methylene (CH_2_) groups. The stretching vibration peak of C–N is observed at 1230 cm^−1^. For the composite gel, it contains both the structure of the supramolecular gel and the polymer gel. In the infrared spectrum, at 1020 cm^−1^ and 550 cm^−1^, the two characteristic peaks of ring vibration and skeleton vibration of the glycosidic bonds of cyclodextrin are distinct. At 1600 cm^−1^ and 1415 cm^−1^, the stretching vibration peaks of carbonyl groups and the bending vibration peak of N–H of amide group are still obvious. At 1440 cm^−1^, the vibration peak of methylene groups is obvious. At 1180 cm^−1^, the stretching vibration peak of C–N is obvious. These show the formation of a composite gel.

**Figure 5:**
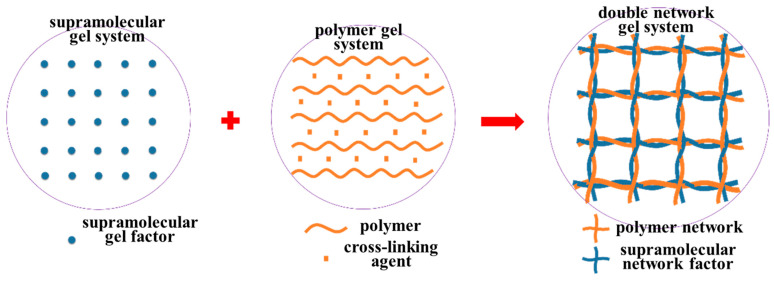
Schematic diagram of the double network structure of the supramolecular–polymer composite gel system.

The root cause is that cyclodextrins can form a supramolecular network structure through self-assembly under the action of polarity regulators, and the polymer and the crosslinking agent can form a polymer network structure through chemical cross-linking. The supramolecular network interweaves with the polymer network, forming a supramolecular–polymer double network system. The schematic diagram of the dual double network structure of the supramolecular–polymer composite gel system is shown in Figure 5. The double network structure can greatly improve the comprehensive performance of the gel.

### 2.3. Rheological Characteristics of the Supramolecular–Polymer Composite Gel System

**Figure 6:**
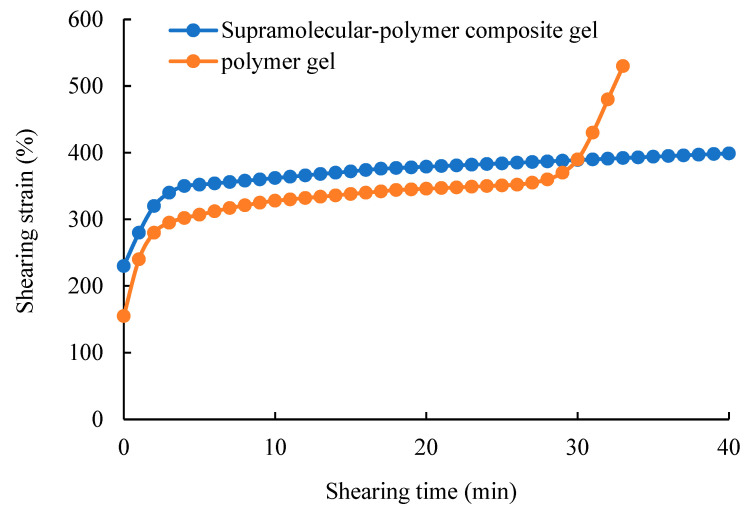
Rheological characteristics of the different types of gels under shearing.

The rheological characteristics of the supramolecular–polymer composite gel and the polymer gel under continuous action at the shearing rate of 100 s^−1^ are shown in Figure 6. Gel rheology refers to the deformation and flow of the gels under shearing. For the polymer gel, it is a single covalently cross-linked network junction, which is a traditional polymer material, and its rheological properties conform to the rheological characteristics of classical polymer materials [41]. After stress is applied to the polymer gel, an instantaneous elastic modulus can appear, and then the strain acceleration stage can appear under the shearing action. Thus, the strain flattening stage can commence. When this action lasts for 30 min, the rapid strain stage can begin. At the rapid strain stage, the polymer gel can be damaged and fractured. For the supramolecular–polymer composite gel, its rheological characteristics are basically the same as that of the polymer gel in the first 30 min of shearing. However, when the polymer gel enters the rapid strain stage, the supramolecular polymer composite gel still stays in the gentle strain stage, indicating that it has a stronger ability to resist shearing. The main reason is that the supramolecular–polymer composite gel contains both the covalent cross-linked network structure and reversible physical cross-linked supramolecular network. Supramolecular networks can be temporarily disconnected under shearing to release the shear stress and keep the overall structure of the gel stable. When the shearing effect is no longer present, the supramolecular network can aggregate again to form a network structure.

### 2.4. Flow Ability of the Supramolecular–Polymer Composite Gel System at the Initial State in Micro-Scale Fractures

**Figure 7:**
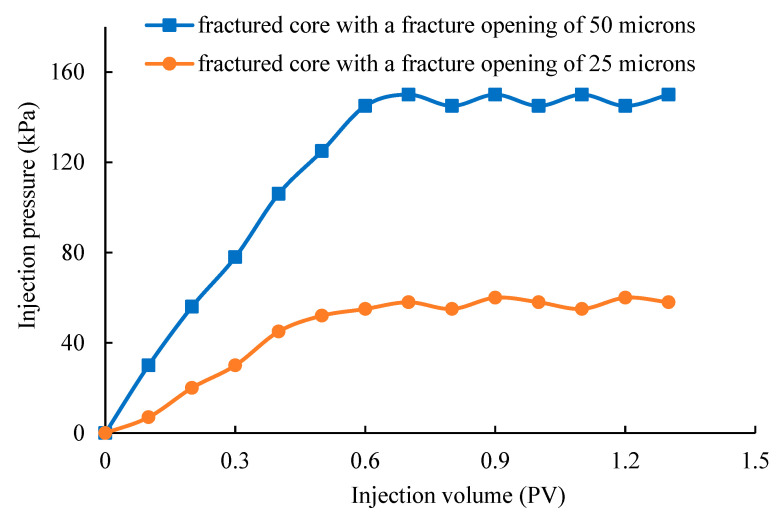
Variation curve of injection pressure with injection amount of the composite gel system at the initial state in micro-fractures.

The results of the composite gel system at the initial state in the micro-scale fractures are shown in Figure 7. From Figure 7, it can be observed that the stable injection pressure of the composite gel system at the initial state in the fracture with the opening of 25 micron is 150 kPa. The injection pressure gradient is 430 kPa/m. The stable injection pressure of the composite gel system at the initial state in the fracture with the opening of 50 micron is 58 kPa. The injection pressure gradient is 166 kPa/m. These show that the composite gel system at the initial state has low injection pressure and can smoothly enter micro-scale fractures, thus entering the deep part of the fractured reservoir.

### 2.5. Plugging Performance of the Gels in the Large Millimeter-Scale Fractures after Gelation

**Figure 8:**
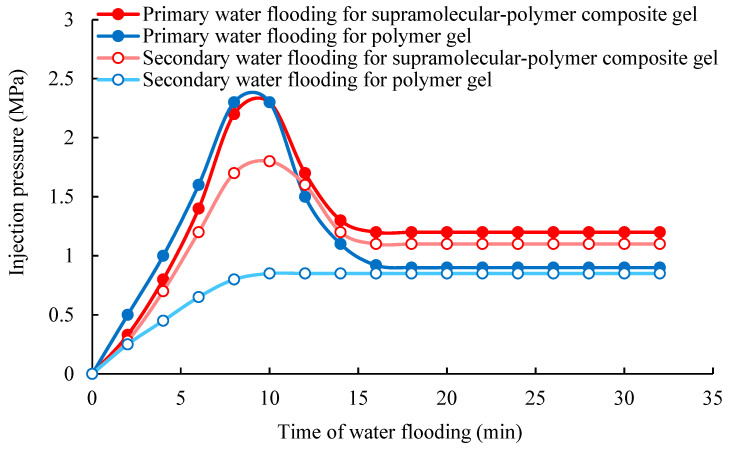
Plugging characteristics of the gels in the core with a 1 mm opening fracture.

**Figure 9:**
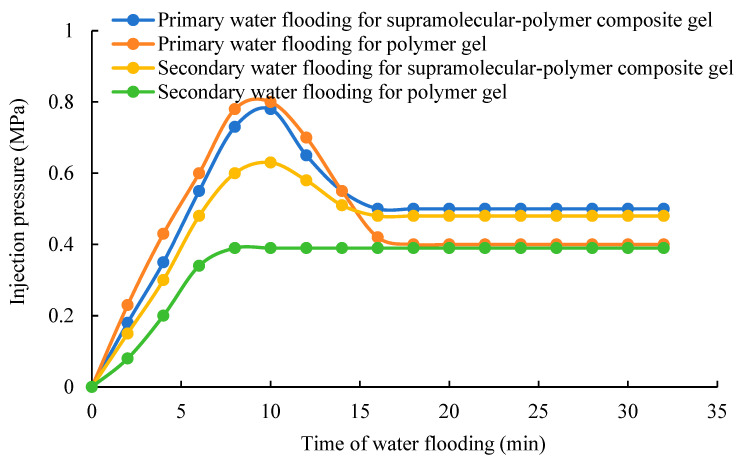
Plugging characteristics of the gels in the core with a 2 mm opening fracture.

After complete gelation, the plugging results of the supramolecular–polymer composite gel and the polymer gel in the large millimeter-scale fractures are shown in Figure 8 and Figure 9. The highest point of injection pressure in the curve is the breakthrough pressure of injected water. During the process of primary water flooding, from Figure 8, it can be observed that the pressure of injected water breaking through the composite gel and the polymer gel in the 1 mm large fracture are both about 2.3 MPa. The breakthrough pressure gradient is about 6.6 MPa/m. From Figure 9, it can be observed that the pressure of the injected water breaking through the composite gel and the polymer gel in the 2 mm large fracture are both about 0.8 MPa. The breakthrough pressure gradient is about 2.3 MPa/m. However, after the injected water breaks through the gels, the injection pressure changes. For the 1 mm large fracture, after the primary water flooding is stable, the plugging pressure of the composite gel is 1.2 MPa, and the plugging pressure of the polymer gel is 0.9 MPa. For the 2 mm large fracture, after the primary water flooding is stable, the plugging pressure of the composite gel is 0.5 MPa, and the plugging pressure of the polymer gel is 0.4 MPa. This shows that after the breakthrough of injected water, the plugging pressure of the composite gel is higher than that of the polymer gel. The reason is that although the strength of the composite gel and the polymer gel are basically the same, the supramolecular network is reversible, and it can be aggregated again after it is broken through. It ensures a dynamic balance to avoid the formation of a new water flow channel. However, the polymer gel is a single chemical cross-linking, which cannot be re-crosslinked after being broken through to form a new water flow channel.

In addition, during the process of the secondary water flooding, the characteristics of the change of pressure of the injected water breaking through the composite gel and the polymer gel are obviously different. For the composite gel, there is a maximum point on the injection pressure curve, and then the pressure begins to decline until it becomes stable. For the polymer gel, the injection pressure gradually rises until it is stable, and there is no highest point. The reason is that during the process of standing for 24 h after the primary water flooding, the supramolecular structure of the composite gel has formed a relatively intact structure through re-crosslinking. When the water is injected again, the injected water needs to break through the network structure again and move forward, so the pressure can rise until the breakthrough pressure is reached. However, because of the irreversibility of the polymer gel structure, its structure cannot be repaired even after standing for 24 h, so the injected water can move forward along the flow channel of the primary water flooding until the pressure remains stable.

## 3. Conclusions

In this paper, a novel supramolecular–polymer composite gel is studied, which consists of a mass fraction of 0.15% of polymer, 0.2% of polyethyleneimine, 1% of cyclodextrin, and 0.2% of ethanol. The composite gel system has excellent injection performance at the initial state, especially in micro-scale fractures with extremely low flow resistance. Subsequently, after gelation, it shows high plugging strength, especially in large-scale fractures with high breakthrough pressure gradient. Under the temperature of 40 °C, the gel system forms a double network structure through supramolecular self-assembly and polymer chemical crosslinking. There are both covalent crosslinking networks and supramolecular networks based on physical crosslinking, which significantly improves the mechanical strength of the gel. These characteristics give it significant advantages in oilfield development, especially in water plugging and oil enhancement of complex fractured reservoirs.

## 4. Experiment

### 4.1. Materials and Instruments

The selected polymer is polyacrylamide with a molecular weight of 6 million Dalton and a degree of hydrolysis of 22%. It is purchased from Changqing oilfield chemical Group Co., Ltd. (Xi’an, China). The cross-linking agent is polyethyleneimine, a commercially available product from Wuhan Biotechnology Co., Ltd. (Wuhan, China). The supramolecular gel factor is cyclodextrin, which is a commercially available product from Hubei Chemical Technology Co., Ltd. (Wuhan, China). The polarity regulator is ethyl alcohol, an analytical grade product from China National Pharmaceutical Group Chemical Reagent Co., Ltd. (Shanghai, China). The water used to prepare the solution in this experiment is tap water.

**Figure 10:**
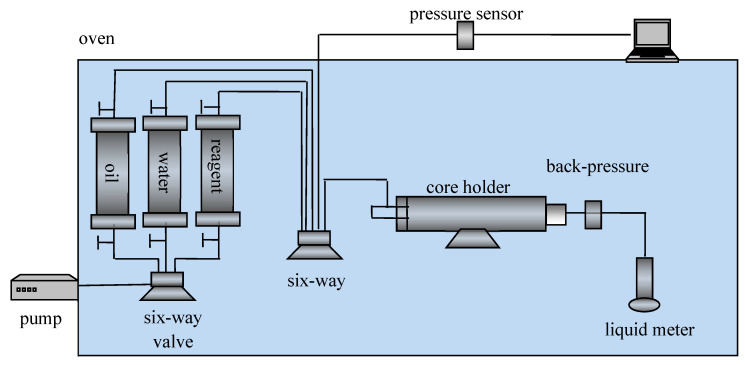
Core displacement experiment device.

The used experiment instruments include Brookfield viscometer, Brookfield company (Middleboro, MA, USA); RS-600HAAKE rheometer, ThermoFisher Scientific company (Waltham, MA, USA); Nicolet 6700 Fourier Transform Infrared Spectrometer (FTIR), Thermo Fisher Scientific company (Waltham, MA, USA); and Physical simulation displacement experimental apparatus, Hai’an Petroleum Research Instruments Co., Ltd. (Hai’an, China) in China. The parameters of the fractured cores are shown in Table 1. The physical simulation displacement experiment apparatus is shown in Figure 10. The inlet of the core holder is connected to a pressure sensor, and the inlet pressure is automatically recorded by the computer. The outlet of the core holder is connected to the atmosphere. The pressure difference (ΔP) at both ends of the core holder is numerically equal to the inlet pressure. The temperature of the displacement experiment is 40 °C, and the injection rate of the fluid is 0.1 mL/min.

### 4.2. Experiment Methods

#### 4.2.1. Preparation and Performance Test of Gels

(1) Preparation and performance test of the supramolecular gel

First, 10 g of cyclodextrin is added to 988 g of water and is dissolved thoroughly under stirring. After sufficient dissolution, 2 g of ethyl alcohol is added to the solution. Next, the viscosity of the solution is measured by the viscometer at a shearing rate of 7.34 s^−1^ after thorough stirring. Then, it is placed into a constant temperature box at 40 °C. After 6 h of gelation, it is taken out. Its viscosity is measured by the viscometer at a shearing rate of 7.34 s^−1^ again and its viscoelasticity is measured by the rheometer at a shearing rate of 0.1 s^−1^ to 100 s^−1^. In the end, the infrared spectrum of the supramolecular gel is tested by the Fourier Transform Infrared Spectrometer.

(2) Preparation and performance test of the polymer gel

First, 1.5 g of polyacrylamide is added to 988 g of water and stirred slowly and uniformly for 120 min until it is fully dissolved. Next, 2 g of polyethyleneimine is added to the solution. Thus, after the solution is fully stirred, its viscosity is measured by the viscometer at a shearing rate of 7.34 s^−1^. After that, the solution is placed into a constant temperature box at 40 °C. After 10 h of gelation, it is taken out. Its viscosity is measured by the viscometer at a shearing rate of 7.34 s^−1^ again and its viscoelasticity is measured by the rheometer at a shearing rate of 0.1 s^−1^ to 100 s^−1^. In the end, the infrared spectrum of the polymer gel is tested by the Fourier Transform Infrared Spectrometer.

(3) Preparation and performance test of the supramolecular–polymer composite gel

First, 1.5 g of polymer is added to 988 g of water and stirred slowly and uniformly for 60 min. After the solution is fully dissolved, 2 g of polyethyleneimine is added to the solution. After the solution is fully stirred again, 10 g of cyclodextrin and 2 g of ethanol are successively added to the solution and stirred thoroughly. Next, the viscosity of the solution is measured at a shearing rate of 7.34 s^−1^. Then, it is placed in a constant temperature box at 40 °C. After 10 h of gelation, it is taken out. Its viscosity is measured by the viscometer at a shearing rate of 7.34 s^−1^ again and its viscoelasticity is measured by the rheometer at a shearing rate of 0.1 s^−1^ to 100 s^−1^. In the end, the infrared spectrum of the supramolecular–polymer composite gel is tested by the Fourier Transform Infrared Spectrometer.

Secondly, to evaluate the shearing resistance of the gel, the characteristics of the strain versus the shearing time of the composite gels under the condition of a constant shear rate of 100 s^−1^ are observed by using the rheometer. Besides, in order to compare the different gels, the strain characteristics of the polymer gel and shearing time are also tested under the same conditions.

Finally, the supramolecular–polymer composite gel sample is freeze-dried, and its microstructure is observed using a scanning electron microscope (SEM). The acceleration voltage is 10 kV, and the amplification factor can be adjusted as needed. Besides, for comparison, the microstructure of the supramolecular gel and polymer gel are also tested using a SEM.

#### 4.2.2. Evaluation of Injection Performance of Conformance Control Agent at the Initial State

The fractured cores 1 # and 2 # containing the micro-scale fractures are selected for the core flooding experiment. The developed supramolecular–polymer composite gel system is taken as the object of evaluation. The injection performance of the supramolecular–polymer composite gel system at the initial state in the micro-scale fractures is tested using the core displacement experiment device at 40 °C. The injection rate during the testing process is 0.1 mL/min, and the injection is continued until the injection pressure is stabilized.

#### 4.2.3. Evaluation of Plugging Performance of Conformance Control Agent after Gelation

The fractured cores 3 # and 4 # containing the large-scale fractures are selected for the core flooding experiment. The developed supramolecular–polymer composite gel system is taken as the object of evaluation. After gelation, the plugging performance of the supramolecular–polymer composite gel in the large-scale fractures is tested by using the core displacement experiment device at 40 °C. First, 2.5 mL of the supramolecular–polymer composite gel system at the initial state is injected into the fractured core 3 #. Next, 5 mL of the supramolecular–polymer composite gel system at the initial state is injected into the fractured core 4 #. Thus, the core holder is closed for 10 h at 40 °C. After the cross-linking reaction and self-aggregation is fully complete, the water is injected into the two fractured cores, respectively. The injection rate is 0.1 mL/min during the testing process. The injection is continued until the injection pressure is stabilized. Subsequently, the injection experiment is stopped and the fractured cores in the core holders are standstill for 24 h. Next, the water is injected into the fractured cores 3 # and 4 # again until the injection pressure is stabilized again. The pressure changes are recorded during the displacement process.

Meanwhile, in order to investigate the difference of plugging characteristics of the supramolecular–polymer composite gel and the polymer gel, two additional groups of comparison experiment are conducted. A total of 2.5 mL of the polymer gel system at the initial state is injected into the fractured core 3 # and 5 mL of the polymer gel system at the initial state is injected into the fractured core 4 #. It should be noted that, in this experiment, the concentration of the composition of the used polymer gel has changed. Here, 8 g of polymer and 2 g of polyethyleneimine are added to 988 g of water, respectively. The initial viscosity of this composition polymer gel system is 1600 mPa·s, and the viscosity after gelation is 55,000 mPa·s at 40 °C. The viscosity after gelation of the polymer gel is basically consistent with that of the developed supramolecular–polymer composite gel, which is conducive to comparison. All other experimental conditions and operations remain consistent with those of the supramolecular–polymer composite gel system. The pressure changes are recorded during the displacement process.

## Figures and Tables

**Table 1:** Parameters of fracture cores.

No.	Length (cm)	Diameter (cm)	Fracture Aperture (mm)	Permeability of Rock Matrix for the Fracture Cores (10^−3^ μm^2^)	Porosity of Rock Matrix for the Fracture Cores (%)
1#	10	2.5	0.025	15	15.66
2#	10	2.5	0.050	15	15.82
3#	10	2.5	1.00	15	15.58
4#	10	2.5	2.00	15	15.64

## Data Availability

The data presented in this study are available on request from the corresponding author.
